# Hypoacylated LPS from Foodborne Pathogen *Campylobacter jejuni* Induces Moderate TLR4-Mediated Inflammatory Response in Murine Macrophages

**DOI:** 10.3389/fcimb.2018.00058

**Published:** 2018-02-27

**Authors:** Kirill V. Korneev, Anna N. Kondakova, Ekaterina N. Sviriaeva, Nikita A. Mitkin, Angelo Palmigiano, Andrey A. Kruglov, Georgy B. Telegin, Marina S. Drutskaya, Luisa Sturiale, Domenico Garozzo, Sergei A. Nedospasov, Yuriy A. Knirel, Dmitry V. Kuprash

**Affiliations:** ^1^Engelhardt Institute of Molecular Biology, Russian Academy of Sciences, Moscow, Russia; ^2^Department of Immunology, Biological Faculty, Lomonosov Moscow State University, Moscow, Russia; ^3^Zelinsky Institute of Organic Chemistry, Russian Academy of Sciences, Moscow, Russia; ^4^CNR Institute for Polymers Composites and Biomaterials, Catania, Italy; ^5^Belozersky Institute of Physico-Chemical Biology, Lomonosov Moscow State University, Moscow, Russia; ^6^German Rheumatism Research Center, Leibniz Institute, Berlin, Germany; ^7^Branch of Shemyakin-Ovchinnikov Institute of Bioorganic Chemistry, Russian Academy of Sciences, Pushchino, Russia

**Keywords:** LPS, lipid A, acyl chains, *Campylobacter jejuni*, pathogenic bacteria, TLR4, proinflammatory cytokines, macrophages

## Abstract

Toll-like receptor 4 (TLR4) initiates immune response against Gram-negative bacteria upon specific recognition of lipid A moiety of lipopolysaccharide (LPS), the major component of their cell wall. Some natural differences between LPS variants in their ability to interact with TLR4 may lead to either insufficient activation that may not prevent bacterial growth, or excessive activation which may lead to septic shock. In this study we evaluated the biological activity of LPS isolated from pathogenic strain of *Campylobacter jejuni*, the most widespread bacterial cause of foodborne diarrhea in humans. With the help of hydrophobic chromatography and MALDI-TOF mass spectrometry we showed that LPS from a *C. jejuni* strain O2A consists of both hexaacyl and tetraacyl forms. Since such hypoacylation can result in a reduced immune response in humans, we assessed the activity of LPS from *C. jejuni* in mouse macrophages by measuring its capacity to activate TLR4-mediated proinflammatory cytokine and chemokine production, as well as NFκB-dependent reporter gene transcription. Our data support the hypothesis that LPS acylation correlates with its bioactivity.

## Introduction

Toll-like receptors constitute a family of immune sensors that recognize conserved molecular patterns associated with bacteria and viruses, mediate interaction of the host immune system with commensal microbiota and initiate early responses to infection (Akira and Hemmi, 2003; Rakoff-Nahoum et al., 2004). Toll-like receptor 4 (TLR4) is critical for effective resistance to Gram-negative bacterial pathogens in mice and humans (Poltorak et al., 1998; Arbour et al., 2000). TLR4 is mainly expressed by myeloid cells such as monocytes, dendritic cells and macrophages (Vaure and Liu, 2014). Activation of TLR4 signaling pathways and downstream transcription factors of NFκB and IRF families leads to production of proinflammatory cytokines and reactive oxygen species (Akira and Takeda, 2004; Lu et al., 2008). These agents can be harmful both to pathogens and to the host cells; indeed, TLR4 involvement has been reported for such pathologies as sepsis, autoimmune diseases and cancer (Cario and Podolsky, 2000; Bank et al., 2014; Korneev et al., 2017).

Lipid A is a biologically active part of LPS, which is responsible for triggering antibacterial immunity (Lüderitz et al., 1978). Stimulation of immune cells with LPS generally occurs via TLR4 that forms a complex with lipid A and extracellular adapter protein MD-2 (Shimazu et al., 1999). Examination of several LPS:TLR4:MD-2 complexes structurally characterized to date (Park et al., 2009; Oblak and Jerala, 2015) clearly suggests that various structural components of lipid A may affect the overall biological activity of LPS to a different extent. The great variety of LPS forms present in nature, particularly in pathogenic bacteria, would then result in a broad spectrum of host responses to LPS. For example, our previous studies showed that the biological activity of LPS purified from several pathogenic bacterial strains was mostly defined by the acylation status of their lipid A (Korneev et al., 2014, 2015). Interspecies structural variations in the components of the TLR4 signaling complex may also influence the efficiency of immune response to LPS, as exemplified by the tetraacylated lipid IVa from *Escherichia coli* which acts as TLR antagonist in human macrophages, while producing a significant response in murine cells (Kovach et al., 1990; Ohto et al., 2012). Pathogenic bacteria can modify their lipid A structure in order to avoid proper recognition by TLR4. For instance, *Y. pestis* produces highly active hexaacyl lipid A in fleas at 25°C, but alters it to a less active tetraacyl form after infecting mammals with higher body temperature of 37°C (Knirel et al., 2005).

*Campylobacter jejuni* is a Gram-negative microaerophilic, flagellate, spiral bacterium, which is the most widespread bacterial cause of human gastroenteritis, accounting for 5–14% of all diarrheal diseases throughout the world (Rautelin and Hänninen, 2000; Young et al., 2007). Infection usually occurs through direct contact with pets or consumption of contaminated products of poultry or cattle, for which *C. jejuni* is a part of normal microbiota (Stephenson et al., 2013). *Campylobacter* infection is considered a mild disease, but it may lead to complications ranging from bacteremia, peritonitis, pancreatitis and hepatitis to miscarriage and autoimmune manifestations, such as arthritis and Guillain-Barré syndrome (Peterson, 1994; Rees et al., 1995; Pope et al., 2007; Fernández-Cruz et al., 2010). Moreover, complications from *Campylobacter* infection can cause death in young, elderly, and immunosuppressed patients, especially in developing countries, making it extremely important from the healthcare perspective (Barton Behravesh et al., 2011).

In this study we purified LPS from a *C. jejuni* O2A strain, determined its composition and assessed its bioactivity in murine macrophages. LPS from *C. jejuni* turned out to be less potent TLR4 activator as compared to LPS from *E. coli*, indicating that the number of acyl chains rather than their length determine LPS bioactivity.

## Materials and methods

### Bacterial cultures and isolation of LPS

The bacterial strains of *Escherichia coli* O130 (Perepelov et al., 2007), *Francisella tularensis* 15 (Mokrievich et al., 2010) and *Campylobacter jejuni* O2A (Moran et al., 1991) were grown as previously described. *E. coli* and *C. jejuni* cells were cultivated under Biosafety Level II (BSL-II) conditions, *F. tularensis* cells was cultivated under BSL-III conditions, according to the Russian Sanitary Regulations SP 1.3.3118-13 and SP 1.3.2322-08 on “Safe handling of microorganisms in pathogenic hazard groups,” approved by decree No. 64, November 28, 2013 and decree No. 4, January 28, 2008 of the Chief State Sanitary Physician of the Russian Federation. LPS from bacterial biomass was purified as previously described (Korneev et al., 2015). Briefly, the biomass was acetone-dried (Robbins and Uchida, 1962), frozen at −70°C, lyophilized and subjected to phenol-water extraction (Jann et al., 1965). R-form LPS was purified by AcA 44 Ultrogel chromatography as described (Korneev et al., 2015). LPS-containing fractions were pooled, desalted by dialysis and lyophilized.

### Mass spectrometry of LPS

MALDI-TOF mass spectrometry of purified LPS samples was performed on a 4800 Proteomic Analyzer (ABSciex, USA), as described (Sturiale et al., 2011). Negative ion mass spectra were acquired in reflector modes with mass accuracy ca. 50 ppm. 2′,4′,6′-Trihydroxyacetophenone monohydrate was used for matrix preparation. Mass spectra were analyzed as described (Sturiale et al., 2011).

### Laboratory animals

All mice were housed under specific pathogen free conditions on 12 h light/dark cycle at 20–23°C and used at the age of 8–10 weeks (weight of 20–22 g). C57Bl/6 mice and *Tlr4*-deficient mice were housed in the Pushchino Animal Breeding Facility (Branch of the Shemyakin and Ovchinnikov Institute of Bioorganic Chemistry, Russian Academy of Sciences). MyD88-deficient mice (Kleinridders et al., 2009) were from the animal facility of the German Rheumatism Research Center (DRFZ), Berlin. All animal manipulations were performed according to recommendations of the Guide for the Care and Use of Laboratory Animals (National Research Council, 2011), the European Convention for the Protection of Vertebrate Animals Used for Experimental and Other Scientific Purposes, Council of Europe (ETS 123), “The Guidelines for Manipulations with Experimental Animals” (the decree of the Presidium of the Russian Academy of Sciences of April 02, 1980, no. 12000-496) and in accordance with German regulations of animal protection. All animal procedures were approved by Scientific Council of the Engelhardt Institute of Molecular Biology.

### Cultivation and activation of bone marrow-derived macrophages

Murine bone marrow-derived macrophages (BMDM) were generated by flushing the femurs and culturing bone marrow cells for 10 days according to the standard protocol (Muller et al., 1996) in DMEM (Gibco, Gaithersburg, MD, USA) supplemented with 30% conditioned medium from L929 cells (a source of M-CSF) and 20% horse serum (Biological Industries, Kibbutz, Israel, lot No. 1630708). To determine the mRNA levels of the cytokines, BMDM were seeded on 12-well plates (10^6^ cells/ml) and treated with LPS species (10 ng/ml) for 2 h. To assess cytokine production in the supernatants, BMDM were stimulated in 96-well plates (10^6^ cells/ml) with LPS (10 ng/ml) for 5 h.

### Real-time quantitative RT-PCR analysis

Total RNA from macrophages was isolated using the TRIzol Reagent (Invitrogen, Carlsbad, CA, USA) according to the manufacturer's protocol. Reverse transcription was carried out using 1.5 mcg total RNA and oligo(dT)_18_ primers with RevertAid First Strand cDNA Synthesis Kit (Thermo Scientific, Waltham, MA, USA) according to manufacturer's protocol. Real-time quantitative PCR was performed using qPCRmix-HS SYBR+LowROX Kit (Evrogen, Moscow, Russia) on the ABI 7500 Real-Time PCR System (Applied Biosystems, Foster City, CA, USA). RT-PCR analysis was performed as described previously (Korneev et al., 2015). The following primers were used: IL-6, 5′-CTC TGC AAG AGA CTT CCA TCC, 5′-TTC TGC AAG TGC ATC ATC GT; TNF, 5′-TCT GTC TAC TGA ACT TCG GG, 5′-TTG GTG GTT TGC TAC GAC; IL-1β, 5′-TCA ACC AAC AAG TGA TAT TCT CCA T, 5′-ACT CCA CTT TGC TCT TGA CTT CT; RANTES, 5′-CCC TCA CCA TCA TCC TCA C, 5′-CCT TCG AGT GAC AAA CAC GA; IP-10, 5′-AAG TGC TGC CGT CAT TTT CT, 5′-GTG GCA ATG ATC TCA ACA CG; IRF3, 5′-AAC CGG AAA GAA GTG TTG CG, 5′-GCA CCC AGA TGT ACG AAG TC and β-actin, 5′-GAC CTC TAT GCC AAC ACA GT, 5′-AGA AAG GGT GTA AAA CGC AG.

### ELISA assay

IL-6 and TNF levels in cell-culture supernatants were determined using a Mouse IL-6 ELISA Ready-SET-Go and Mouse TNF alpha ELISA Ready-SET-Go kits (eBioscience, San Diego, CA, USA) according to the manufacturer's instructions.

### Luciferase reporter assay in RAW264.7 cell line

NFκB-responsive luciferase reporter construct containing minimal CMV promoter and five tandem copies of NFκB consensus site have been described previously (Mitkin et al., 2015). Luciferase reporter vector pmIL-6 FL containing full-length promoter of murine IL-6 gene was a gift from Gail Bishop (Addgene plasmid # 61286) (Baccam et al., 2003).

The murine macrophage-like cell line RAW264.7 was maintained in Dulbecco's modified Eagle medium (DMEM, Life technologies, Carlsbad, CA, USA) with 4.5 g/l glucose. Culture medium was supplemented with 10% fetal bovine serum (Biological Industries, Kibbutz, Israel, lot No. 1540726), 2 mM L-glutamine, 1 mM sodium pyruvate, 100 U/ml penicillin and 100 mcg/ml streptomycin, MEM non-essential amino acids and 10 mM HEPES (all, Gibco, Gaithersburg, MD, USA). Cells were transfected with 5 mcg of purified plasmid DNA and 300 ng of pRL-CMV control Renilla luciferase reporter vector (Promega, Madison, WI, USA) using Neon Transfection System (Thermo Scientific, Waltham, MA, USA). All electroporations were carried out with one 20 ms 1900 V pulse using 100 mcl tip with cell density 2 × 10^7^ cells/ml in resuspension buffer T. 24 h after electroporation, cells were treated with LPS preparations (10 ng/ml) for 6 h and then luciferase activity was measured using Dual-Luciferase Reporter Assay System (Promega, Madison, WI, USA) and Luminometer 20/20n (TurnerBioSystems, Sunnyvale, CA, USA) following the manufacturer's instructions. The activity of Firefly luciferase was normalized to the activity of Renilla luciferase to account for fluctuations in electroporation efficiency.

### IRF3 knockdown with siRNA

IRF3 knockdown was performed using IRF3-specific and control scrambled siRNA synthesized by Syntol, Moscow, Russia. Sense and antisense single-stranded RNA were annealed by slow cooling down from 95° to 25°C in annealing buffer (10 mM Tris, 20 mM NaCl, pH 8.0). At day 1, RAW264.7 macrophages were electroporated (as described above) with 500 pmol siRNA duplexes. In order to prolong the silencing effect, at day 3 cells were transfected with 300 more pmol of the same siRNA duplexes. At day 5 macrophages were treated with LPS preparations (10 ng/ml) for 2 h to determine the mRNA levels of the proinflammatory cytokines and chemokines. Oligonucleotides used for IRF3 knockdown: IRF3: 5′-AAG GUU GUU CCU ACA UGU CUU dTdT, 5′-AAG ACA UGU AGG AAC AAC CUU dTdT; scrambled: 5′-GUU CUA UCG AUC CUG GAA UUG dTdT, 5′-CAA UUC CAG GAU CGA UAG AAC dTdT.

### Statistical analysis

Statistical analysis was carried out using GraphPad Prism software (version 6, San Diego, CA, USA). All data passed the D'Agostino-Pearson omnibus normality test. One-way ANOVA or two-way ANOVA with Tukey's test were used for multiple pairwise comparisons. The data were obtained in at least three independent experiments and presented as the mean ± SD. *P* < 0.05 were considered to indicate statistical significance.

## Results

### Characterization of lipid a of the *C. jejuni* LPS by MALDI-TOF MS

**Figure 1:**
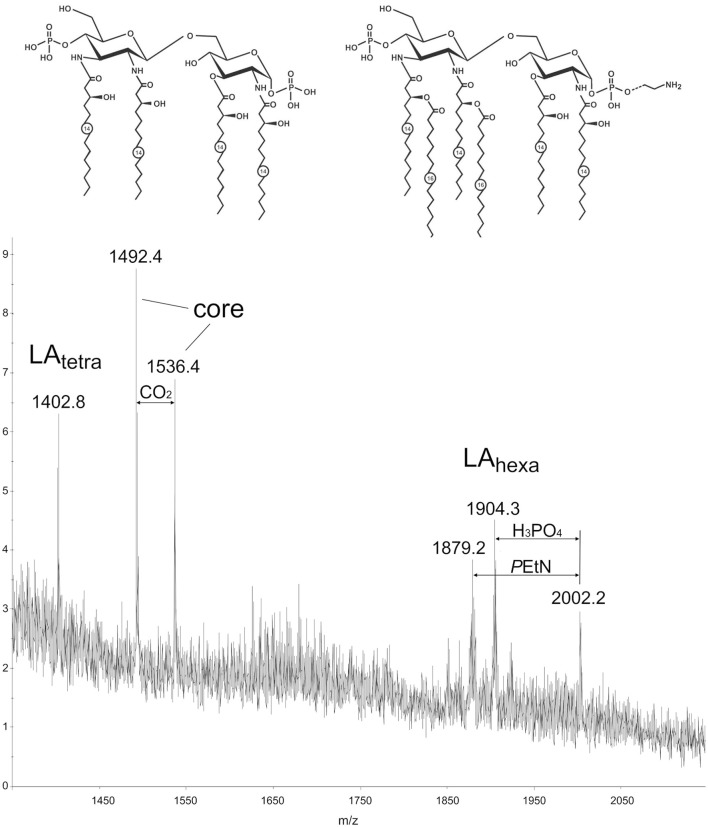
MALDI-TOF mass spectrum of the LPS from a *C. jejuni* O2A strain. Structures of the tetraacyl and hexaacyl lipid A species shown in the inset are based on the structures that have been established by chemical and MS analysis of the isolated lipid A of *C. jejuni* (Moran et al., 1991). Dotted line indicates non-stoichiometric substitution with phosphoethanolamine (PEtN). Numbers indicate the number of carbons in the acyl chain.

Structure of the lipid moiety (lipid A) of the LPS from a *C. jejuni* O2A strain was analyzed by MALDI-TOF MS in the negative ion mode (Figure 1). The mass spectrum shows peaks of the lipid A and core moieties that originated from in-source fragmentation of the LPS (Sturiale et al., 2011). A peak for an Y-type fragment at m/z 1402.8 belongs to a tetraacylated lipid A species (LA_tetra_) having a biphosphorylated hybrid hexosamine disaccharide backbone that consists of one residue each of d-glucosamine and 2,3-diamino-2,3-dideoxy-d-glucose and carries four residues of 3-hydroxymyristic acid. Peaks in a higher mass regions corresponded to hexaacylated species (LA_hexa_) with two additional residues of palmitic acid (m/z 1879.2), some species carrying phosphoethanolamine (m/z 2002.2). These findings are basically in agreement with the structure that has been established by chemical and MS analysis of the isolated lipid A of *C. jejuni* (Moran et al., 1991).

### Stimulation of macrophages with LPS from *C. jejuni* results in an equally moderate activation of all TLR4-mediated proinflammatory signaling pathways

**Figure 2:**
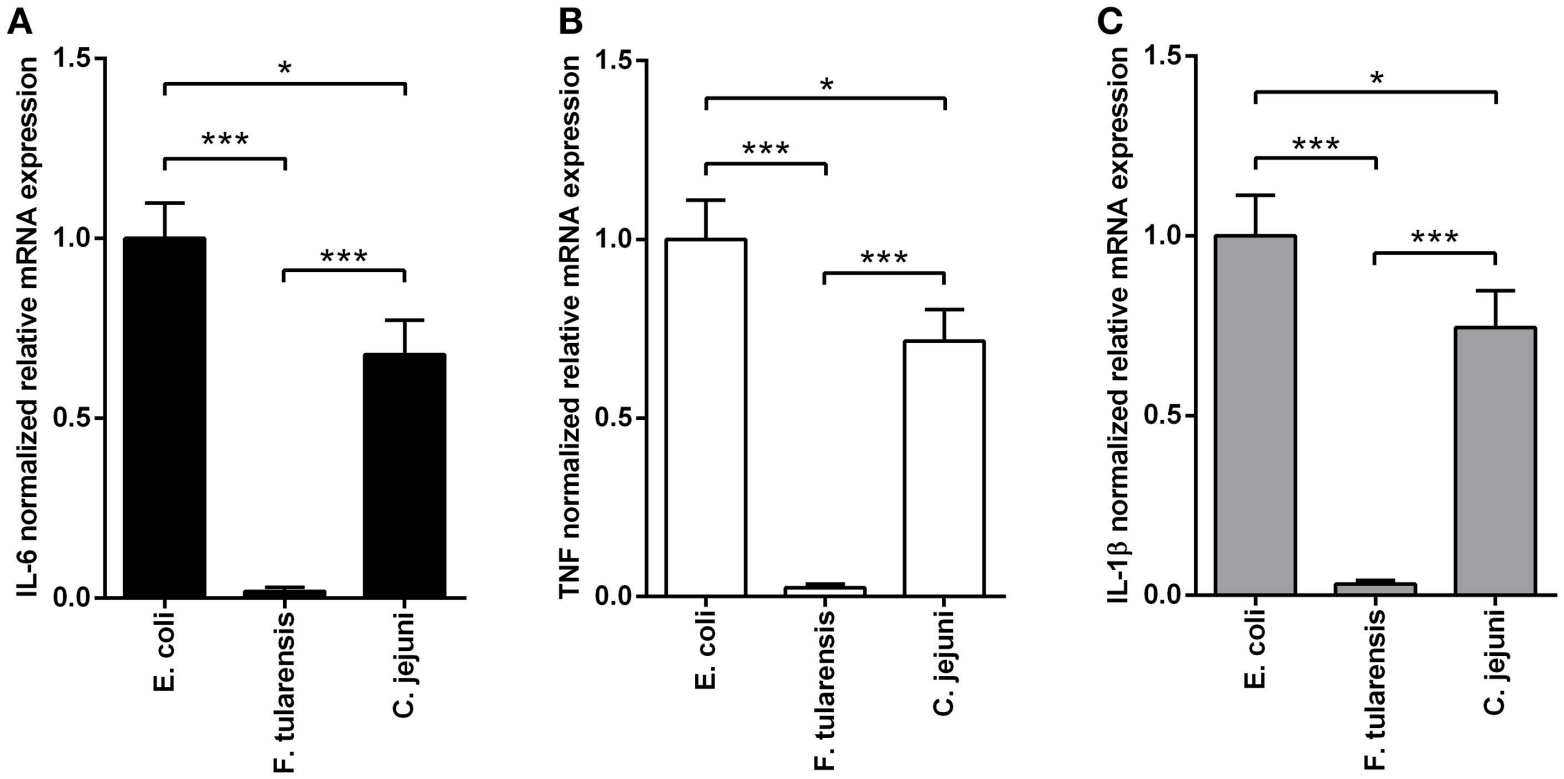
LPS isolated from a *C. jejuni* O2A strain is a mild activator of mRNA expression of proinflammatory cytokines in BMDM. Quantification of IL-6 **(A)**, TNF **(B)**, and IL-1β **(C)** mRNA levels in BMDM from WT mice. Relative mRNA expression levels were normalized to β-actin. All data are representative of five independent experiments. Data represent mean values ± SD. ^*^P < 0.05, ^***^P < 0.001, as calculated by one-way ANOVA with Tukey's test were used for multiple pairwise comparisons.

**Figure 3:**
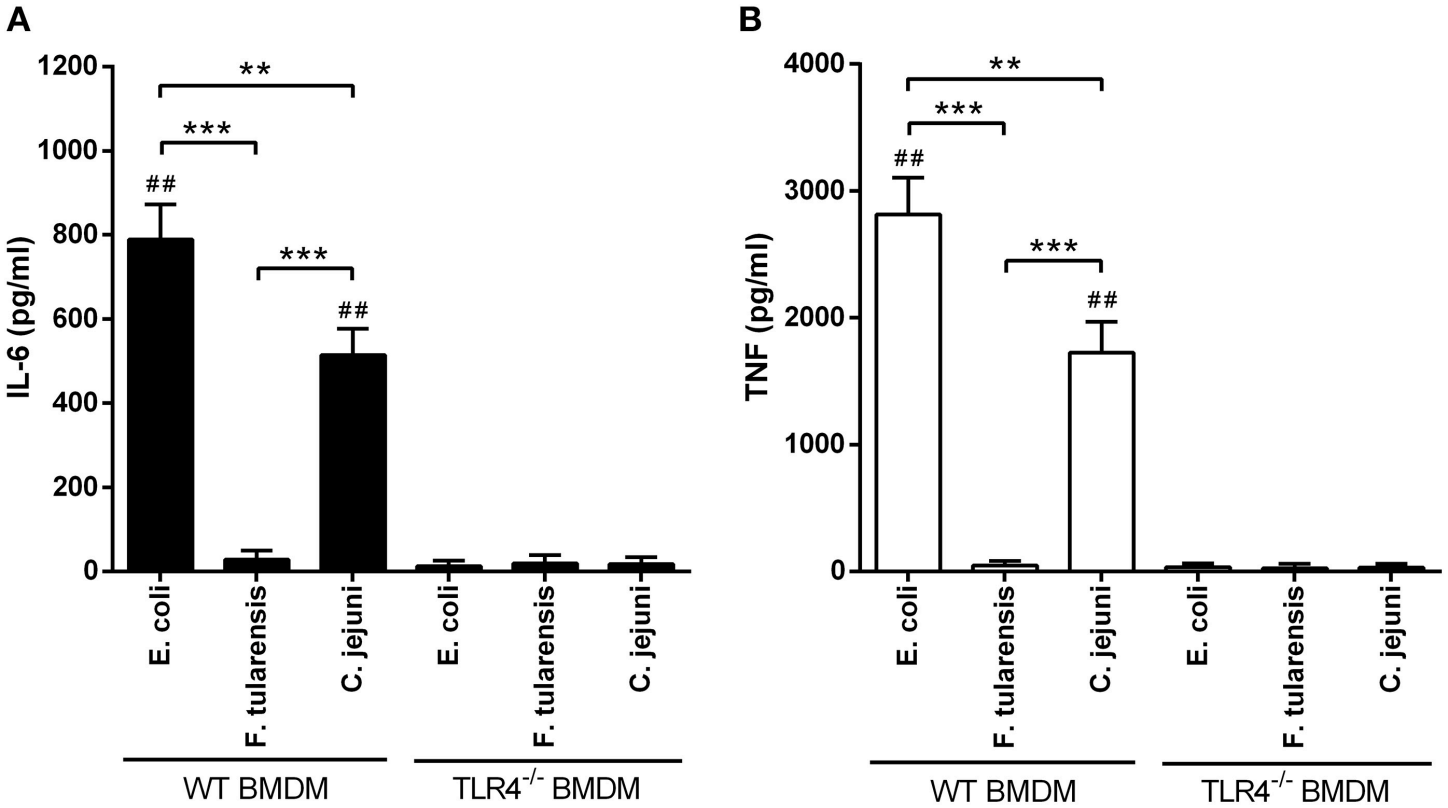
Stimulation of BMDM with LPS from a *C. jejuni* O2A strain leads to lower production of proinflammatory cytokines compared to LPS from *E. coli*. ELISA quantification of IL-6 **(A)** and TNF **(B)** levels in the supernatants of LPS-stimulated BMDM from WT and TLR4^−/−^ mice. LPS preparations did not induce production of proinflammatory cytokines in TLR4^−/−^ BMDM. All data are representative of five independent experiments. Data represent mean values ± SD. ^**^P < 0.01, ^***^P < 0.001, as calculated by two-way ANOVA with Tukey's test for multiple pairwise comparisons. ^##^*P* < 0.001 indicates statistically significant LPS activity on WT BMDM vs. TLR4^−/−^ BMDM.

In order to assess the biological activity of LPS preparations, we measured their ability to induce expression of proinflammatory cytokines in BMDM at the mRNA level 2 h after activation (Figure 2) and at the protein level after 5 h of stimulation (Figure 3). Similarly to other bioactive LPS preparations (Korneev et al., 2015), our assays with LPS from *C. jejuni* demonstrated a dynamic range from 0.1 to 100 ng/ml, therefore a working concentration of 10 ng/ml was used in all subsequent experiments.

We used highly active LPS isolated from *E. coli* with hexaacyl biphosphoryl lipid A (Qureshi et al., 1988) as positive control. In addition, inactive LPS from *F. tularensis* with tetraacyl monophosphoryl lipid A (Vinogradov et al., 2002) was used as negative control. BMDM stimulated with *C. jejuni* LPS showed lower levels of IL-6, TNF and IL-1β gene expression in comparison with LPS from *E. coli* (Figure 2). Specificity of TLR4 recognition of LPS preparations was assessed by stimulating BMDM culture from TLR4-deficient mice (Figure 3).

We then assessed the production of IL-6 and TNF proteins by the BMDM 5 h after LPS treatment. Concentrations of both cytokines in the culture medium very closely followed the pattern of mRNA expression, indicating that the observed results were not due to translational regulation (Figure 3).

**Figure 4:**
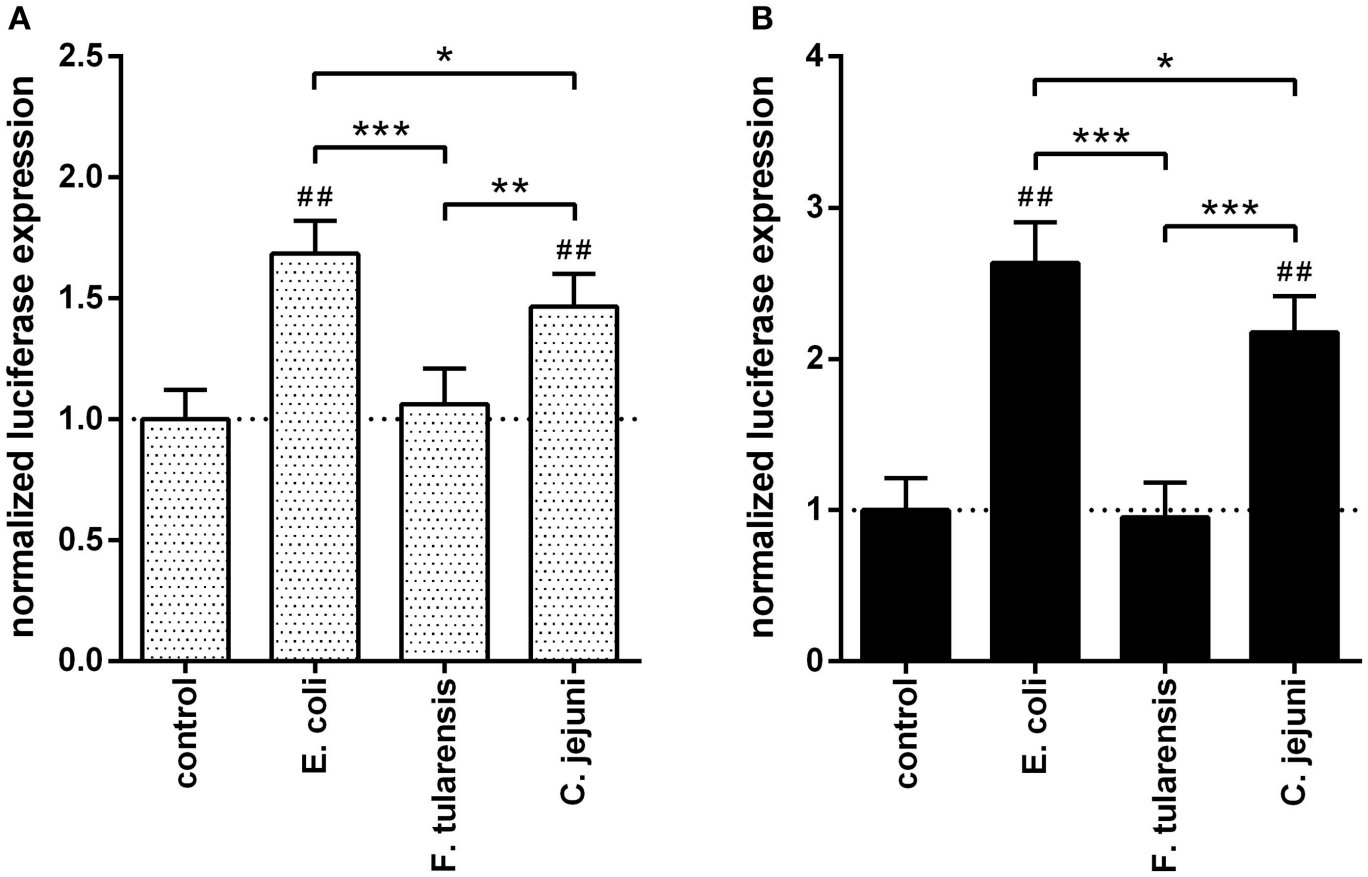
LPS from a *C. jejuni* O2A strain induced a moderate level of NFκB activation in RAW264.7 cells. The bars correspond to the normalized expression levels of luciferase reporter constructs under the control of NFκB-responsive synthetic promoter **(A)** and IL-6 promoter **(B)** in RAW264.7 cells induced by treatment with various LPS preparations. Control group did not receive any treatment with LPS; the dotted lines indicate the baseline. All data are representative of three independent experiments. Data represent mean values ± SD. ^*^P < 0.05, ^**^P < 0.01, ^***^P < 0.001, as calculated by one-way ANOVA with Tukey's test were used for multiple pairwise comparisons. ^##^*P* < 0.001 indicates statistically significant reporter activity of LPS from *E. coli* and *C. jejuni* vs. control.

To further investigate the transcriptional effects of different LPS preparations on TLR4 signaling, we used two NFκB-dependent reporter constructs. One of the constructs contained a luciferase gene under the control of NFκB-responsive synthetic promoter (Figure 4A) and another one employed the murine IL-6 promoter previously shown to contain an NFκB binding site critical for its activity (Baccam et al., 2003; Figure 4B). The RAW264.7 murine macrophage cell line was used instead of BMDM due to its higher transfection efficiency. In both cases, the effects by LPS from *C. jejuni* were moderately but significantly lower as compared to those produced by LPS from *E. coli* (Figure 4).

**Figure 5:**
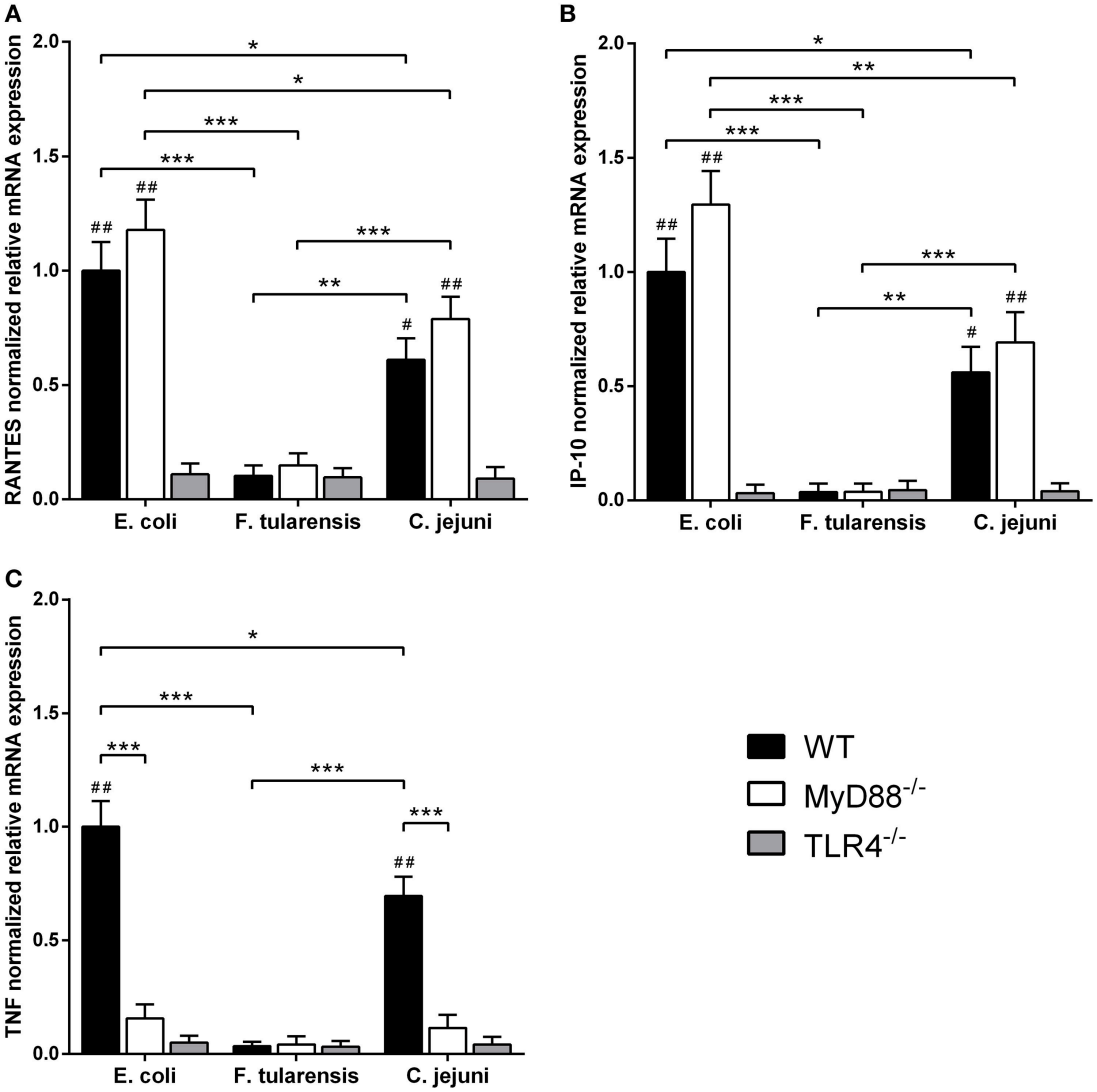
LPS from a *C. jejuni* O2A strain is a moderate inducer of mRNA expression of MyD88-independent proinflammatory chemokines in BMDM. RANTES **(A)**, IP-10 **(B)**, and TNF **(C)** mRNA levels in BMDM isolated from MyD88^−/−^, TLR4^−/−^ and WT mice. Relative mRNA expression levels were normalized to β-actin. All data are representative of three independent experiments. Data represent mean values ± SD. ^*^P < 0.05, ^**^P < 0.01, ^***^P < 0.001, as calculated by two-way ANOVA with Tukey's test for multiple pairwise comparisons. ^#^*P* < 0.01 and ^##^*P* < 0.001 indicates statistically significant LPS activity on WT or MyD88^−/−^ BMDM vs. TLR4^−/−^ BMDM.

**Figure 6:**
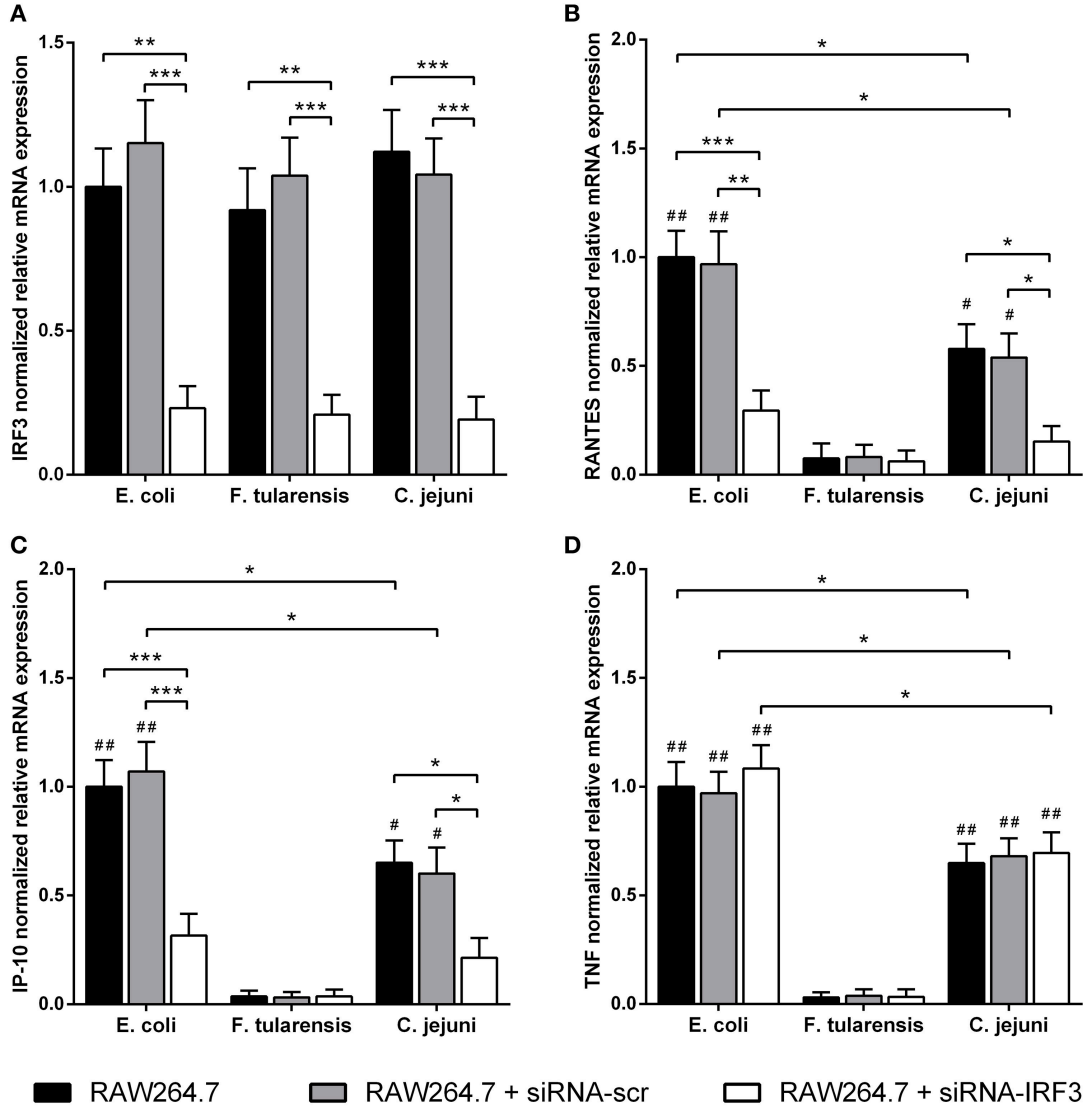
Reduced LPS-mediated mRNA expression of RANTES and IP-10 chemokines after IRF3-knockdown in RAW264.7 macrophages. Quantification of IRF3 **(A)**, RANTES **(B)**, IP-10 **(C)**, and TNF **(D)** mRNA levels in RAW264.7 cells with IRF3-knockdown after treatment with LPS species. Relative mRNA expression levels were normalized to β-actin. All data are representative of five independent experiments. Data represent mean values ± SD. ^*^P < 0.05, ^**^P < 0.01, ^***^P < 0.001, as calculated by two-way ANOVA with Tukey's test for multiple pairwise comparisons. ^#^*P* < 0.01 and ^##^*P* < 0.001 indicates statistically significant LPS bioactivity from *E. coli* or *C. jejuni* vs. LPS from *F. tularensis*.

In order to assess the contribution of MyD88-independent TLR4 signaling in our system, we used BMDM generated from MyD88-deficient mice and measured mRNA levels of interferon-inducible proinflammatory chemokines RANTES and IP-10 encoded by *Ccl5* and *Cxcl10* genes, known targets of the TLR4-TRIF-IRF3 pathway (Lin et al., 1999; Kawai et al., 2001). We observed that the difference in activity between *C. jejuni* and *E. coli* LPS preparations was the same for both MyD88-dependent and -independent TLR4 signaling pathways. Similarly to TNF, IL-6 and IL-1β, activation of RANTES and IP-10 by LPS was completely abolished in TLR4-deficient cells (Figure 5). In order to further demonstrate the lack of cross-coupling of TLR4 signaling pathways, we performed IRF3 knockdown in RAW264.7 cells using siRNA against *Irf3* gene. IRF3-specific siRNA caused at least 5-fold suppression of IRF3 mRNA in RAW264.7 macrophages on day 5 after the first transfection (Figure 6A) and led to a significant decrease in RANTES and IP-10 mRNAs (Figures 6B,C), while the level of TNF mRNA remained unchanged (Figure 6D). These observations suggest that LPS bioactivity depends on its interaction with TLR4 rather than on specific intracellular pathways.

## Discussion

We have previously assessed the bioactivity of LPS preparations from various pathogenic bacteria, such as *Y. pestis* (the cause of plague), *B. mallei* (the cause of glanders and melioidosis), *P. aeruginosa* and *A. baumannii* (the causes of nosocomial infections), as well as from ancient psychrotrophic bacteria *P. cryohalolentis* and *P. arcticus* (Korneev et al., 2014, 2015). The lower length of acyl groups in LPS of *A. baumannii* (C12-14) and *Psychrobacter* spp. (C10-12) as compared to the highly active LPS of *E. coli* (C14) resulted in a weaker bioactivity, indicating that LPS with longer acyl groups is a more robust activator of TLR4 signaling.

Lipid A from a *C. jejuni* O2A strain has on the average longer acyl groups (C14-16) as compared to LPS from *E. coli* (C14), but nevertheless it demonstrates lower biological activity (Figures 2–5). This may be explained by the acylation status of LPS from *C. jejuni* which contains a mixture of tetra- and hexaacylated forms of lipid A, while lipid A from *E. coli* is predominantly hexaacylated. As previously shown, a tetraacylated lipid A is a less potent activator of TLR4 than hexaacylated lipid A with fatty acid residues of the same length (Korneev et al., 2014), and our results with LPS from a *C. jejuni* O2A strain corroborate the concept of the number of acyl chains in lipid A having stronger effect on LPS bioactivity than their length. In addition, this reduction in activity may be explained by the presence of phosphoethanolamine residue on one of the phosphate groups in some of the hexaacylated forms of lipid A (Figure 1), that may partially neutralize the negative charges of the phosphate groups which are essential for the efficient interaction with the positively charged amino acids of TLR4/MD-2 signaling complex (Molinaro et al., 2015; Oblak and Jerala, 2015). Thus, several structural traits of lipid A from *C. jejuni* may have a cumulative effect on moderating the activation of TLR4 signaling.

Even though any given pathogen can usually activate more than one TLR family member, it is TLR4 that is crucial for the recognition of *C. jejuni*. For instance, *C. jejuni* can evade TLR5 recognition by altering amino acid sequence of flagellin (Andersen-Nissen et al., 2005), while TLR4 by itself can provide proper recognition and induce sufficient immune response (Rathinam et al., 2009). Moreover, it was previously shown that strains of *C. jejuni* with modifications of LPS that promote inflammatory reactions are associated with elevated severity of gastroenteritis, suggesting a leading role for TLR4 in activation of innate immunity in response to this pathogen (Mortensen et al., 2009; Kuijf et al., 2010).

TLR4-dependent LPS responses may also be reduced below the threshold required for effective immune response if pathogenic bacteria modify their lipid A structure to a sufficient extent. This may eventually result in the failure of local and systemic bacterial clearance. At the same time, moderation of anti-bacterial responses may be advantageous for infected patients in clinical practice, since such an attenuated LPS may not be able to induce severe sepsis in susceptible individuals (Ramachandran, 2014). Technically challenging fractionation of LPS species by the degree of lipid A acylation was not attempted in this study, however LPS containing a mixture of tetraacyl and hexaacyl forms with moderate bioactivity in murine macrophages is predicted to generate a reduced immune response in humans, as human TLR4 is unable to respond a tetraacyl form of lipid A (Golenbock et al., 1991).

LPS forms from our *C. jejuni* strain is structurally different from various LPS species tested for bioactivity in previous studies (Schromm et al., 2000; Stephenson et al., 2013). Stephenson et al. studied LPS from 15 different *C. jejuni* isolates all of which had 3 or 4 phosphate groups attached to the disaccharide backbone of lipid A (Stephenson et al., 2013). *C. jejuni* lipid A from report by Schromm et al. had two different classes of secondary acyl chains (C16:0 and C14:0) and did not contain any phosphoethanolamine residues. Furthermore, such LPS had predominantly hexaacyl form of lipid A and no tetraacyl fraction (Schromm et al., 2000) that was reliably detectable in our preparations (Figure 1). Our data extend and complement these reports, supporting the hypothesis that LPS preparations with a higher acylated lipid A moiety are better inducers of TLR4-signaling.

A limitation of using LPS from different bacteria for clinical or biological studies of endotoxin activity is its microheterogeneity with regard to lipid A structural components, such as the phosphate residues or the number and length of acyl groups (Matsuura, 2013). Thus, the purification, structural analysis, and biological characterization of LPS from bacteria distinct in their pathogenicity are of considerable interest. Better understanding of relationship between LPS structure and its activity may facilitate novel methods for the fine-tuning of antibacterial immune response.

## Author contributions

KK, MD, LS, DG, SN, YK, and DK designed research. KK, ANK, ES, NM, AP, AAK, and GT performed experiments. KK, DG, SN, YK, and DK wrote the manuscript. All authors analyzed data and contributed to the final version of the manuscript.

### Conflict of interest statement

The authors declare that the research was conducted in the absence of any commercial or financial relationships that could be construed as a potential conflict of interest.
